# The effects of dietary intervention combined with comprehensive exercise instruction on somatic function and quality of life in elderly patients with diabetes mellitus

**DOI:** 10.12669/pjms.40.6.7897

**Published:** 2024-07

**Authors:** Xu Sha, Xiaoxiao Zhao, Yao Chen

**Affiliations:** 1 Xu Sha, Department of Geriatrics, The No.2 Hospital of Baoding, Baoding 071000, Hebei, China; 2 Xiaoxiao Zhao, Department of Geriatrics, The No.2 Hospital of Baoding, Baoding 071000, Hebei, China; 3 Yao Chen, Department of Geriatrics, The No.2 Hospital of Baoding, Baoding 071000, Hebei, China

**Keywords:** Dietary Intervention, Comprehensive Exercise Instruction, Elderly, Diabetes Mellitus

## Abstract

**Objective:**

To assess the effects of dietary intervention combined with comprehensive exercise instruction on somatic function and quality of life in elderly patients with diabetes mellitus (DM).

**Methods:**

This was application research. A total of 120 elderly patients with type-2 diabetes mellitus (T2DM) admitted to The No.2 Hospital of Baoding from March 10, 2022 to March 10, 2023 were included and randomly divided into the control group and the experimental group(n=60). Patients in the control group received conventional treatment and nursing regimen, while those in the experimental group were given dietary intervention combined with comprehensive exercise instruction based on the control group. The differences before and after treatment between the two groups were compared and analyzed.

**Results:**

After the intervention, the experimental group had remarkably lower levels of fasting blood glucose (FBG), 2h postprandial blood glucose(2hPG) and glycated hemoglobin (HbA1c) than the control group (p=0.00), while significantly improved somatic function, psychological function, social function and material life status compared to the control group (p=0.00). The levels of SAS and SDS in the experimental group were significantly decreased compared with those in the control group (p=0.00). The levels of SOD, MDA and CAT in the experimental group were obviously superior to those in the control group, with statistically significant differences (p=0.00).

**Conclusion:**

Dietary intervention combined with comprehensive exercise instruction is an effective treatment for elderly patients with DM, boasting a variety of benefits such as regulating patients’ blood glucose levels, improving patient satisfaction, and ameliorating the level of oxidative stress in the body, which is worthy of clinical promotion.

## INTRODUCTION

Diabetes mellitus (DM), a common chronic disease in internal medicine, is prevalent in middle-aged and elderly people, posing a huge risk to human health. Type-2 diabetes mellitus (T2DM) accounts for up to 95% of all DM cases.^1^ Recent years have witnessed an increasing incidence of DM, which, if left untreated, may lead to a variety of complications that can seriously affect the quality of life of patients and even lead to death.^2^ With a long course of the disease, patients with DM require long-term control of their blood glucose levels. Clinically, DM is preferably treated with drugs to control blood glucose levels to reduce or delay the occurrence of complications. However, there is little scientific evidence to indicate the optimal treatment for T2DM at present.^3^ While medication works, DM patients’ behaviors, self-management ability and dietary intake will also have a greater impact on their own glycemic control outcomes.^4^ In particular, elderly patients with DM who, because of their reduced behavior and understanding, often fail to cooperate effectively with clinical care. For this reason, a targeted, comprehensive and effective nursing intervention is conducive to the control of patients’ condition and blood glucose levels under the cooperation of medication. In addition, proper diet and exercise benefit the glucose and lipid metabolism of DM, thus reducing insulin resistance (IR) and ameliorating lipid levels.^5^ Currently, most elderly patients with DM are trained with a single exercise or on a simple low-sugar diet. In this study, 120 elderly patients with DM were recruited for group study to observe the effects of dietary intervention combined with comprehensive exercise instruction on somatic function and quality of life of such patients, with a view to providing a case basis for clinical treatment.

## METHODS

This was application research. One hundred and twenty elderly patients with T2DM admitted to The No.2 Hospital of Baoding from March 10, 2022 to March 10, 2023 were included and randomly divided into two groups: the control group and the experimental group, with 60 cases in each group. No statistically significant differences were observed between the two groups in terms of general data, indicating the comparability of differences between the groups (Table-I).

**Table-I:** Comparative analysis of general data between the two groups (*χ̅*±*S*) n=60.

Indexes	Experimental group	Control group	t/χ^2^	P
Age (years old)	69.43±4.72	70.83±4.31	1.70	0.10
Male (cases, %)	38 (63%)	35 (58%)	0.31	0.58
Medical history (years)	12.23±6.71	13.03±7.13	0.62	0.53
BMI (kg/m^2^)	23.52±4.07	23.71±4.33	2.45	0.80
Complications				
Hyperlipidemia (cases, %)	15 (25%)	17 (28%)	0.17	0.68
Hypertension (cases, %)	14 (23%)	11 (18%)	0.45	0.50
Coronary artery disease (cases, %)	6 (10%)	5 (8%)	0.10	0.75

p>0.05.

### Ethical Approval:

The study was approved by the Institutional Ethics Committee of The No.2 Hospital of Baoding (No.:2023-015; date: June 15,2023), and written informed consent was obtained from all participants.

### Inclusion criteria:

- Patients who met the diagnostic criteria for type 2 diabetes mellitus (T2DM);^6^
- Patients aged >60 years;
- Patients with no severe complications in other organs;
- Patients who voluntarily participated in the study and signed informed consent;
- Patients with complete clinical data;
- Patients who could cooperate to complete the study;
- Patients who signed informed consent;
- Patients without severe joint lesions;
- Patients who themselves and their family members were informed of this study and signed the consent form.

### Exclusion criteria:

- Patients with acute complications of T2DM;
- Patients with poor basic conditions or extreme weakness;
- Patients with endocrine diseases or vital organ dysfunction;
- Patients who have taken hormones and other drugs for a long time;
- Patients with recent trauma, surgery, acute and chronic infections and other stressful conditions;
- Patients with mental illness, cognitive impairment and communication disorders;
- Patients who dropped out of the study midway.

Patients in the control group received conventional treatment and nursing regimens. Specifically, routine health education was given upon admission to familiarize the patients and their families with the hospital as soon as possible; Moreover, relevant treatments were given under medical prescription, including administrating glucose-lowering drugs by oral or intravenous injection, cognitive interventions, diabetic medication interventions and dietary interventions, and routine physical activities, depending on patients’ condition.

Patients in the experimental group were given dietary intervention combined with comprehensive exercise instruction on top of the control group as follows:

### Individualized dietary intervention:

Upon admission, patients’ blood glucose and body mass index were measured and calculated, and nutritional screening was conducted by experienced dietitians to judge their nutritional status. A personalized comprehensive dietary care regimen was developed for each patient, taking into account their dietary structure, habits, blood glucose test results, physical function, and disease progression. On the one hand, the importance of diet control was explained to patients and their families to clarify the relationship between diet and the onset of T2DM and disease control, and to guide patients and their families to manage the patients’ diet structure. In addition, a consultation hotline was set up to ensure timely and effective guidance for patients in case of questions. For those who were overweight, weight loss was also carried out in conjunction. While supplementing conventional calories, it was ensured that the daily intake of carbohydrates accounted for 50%-60% of the total calories, high-quality protein accounted for 15%-20% of the total protein, and low-fat food accounted for 25%-30% of the total calories.

### Comprehensive exercise instruction:

effective exercise is helpful to consume body heat and reduce body weight. It is clinically recognized as a relatively safe and non-drug therapy without adverse reactions when combined with dietary intervention. Given that this study targeted elderly patients, attention was paid to the physical condition of the patients and the availability of someone to protect them during exercise therapy. Appropriate aerobic exercise was performed one hour after meals to help patients consume calories to a certain extent, which was less likely to cause diabetic hypoglycemia. Depending on the patients’ condition, exercises such as walking were performed and maintained for 30 minutes each time. In case of any fatigue and discomfort, patients should stop exercising immediately and adjust the intensity of the next exercise. Mean follow up period was six months.

### Observation indexes:

Comparative analysis of clinical efficacy: indexes such as fasting blood glucose (FBG), two hours postprandial blood glucose (2hPG) and glycosylated hemoglobin (HbA1c) were taken before and after the intervention, and the differences between them were compared and analyzed;

Comparative analysis of quality of life of the two groups before and after the intervention: the quality of life of patients was assessed using the Generic Quality of Life Inventory-74 (GQOLI-74), including somatic function, psychological function, social function and material life status. The questionnaire consists of 20 questions targeting 74 items, each with a score of one to five. An increase in the score indicates an improvement in the quality of life.^7^

Comparative analysis of emotional status: the Anxiety Self-Assessment Scale(SAS) and the Self-rating Depression Scale(SDS)^8^ were employed to assess the emotional changes of the two groups before and after the intervention, with a lower score indicating a better emotional status;

### Comparative analysis of patient satisfaction:

the Patient Satisfaction Questionnaire Short Form (PSQ-18)^9^ was utilized to compare the patient satisfaction before and after the intervention, including very satisfied, generally satisfied, satisfied, uncertain, and satisfied.

*Comparative analysis of oxidative stress indexes:* 10ml of fasting venous blood was drawn from patients before and after the intervention, and their levels of superoxide dismutase (SOD), malondialdehyde (MDA) and catalase (CAT) were measured.

### Statistical analysis:

All data in this study were statistically analyzed using SPSS 20.0 software, and the measurement data were expressed as (*χ̅*±*S*). Two independent sample *t* test was employed for comparison between the two groups, paired t-test was utilized for intra-group comparison. The confidence interval was 95%. Besides, χ^2^ test was used for comparison between the two groups, with a p<0.05 indicating a statistically significant difference.

## RESULTS

No statistically significant differences were observed in the levels of fasting blood glucose (FBG), two hours postprandial blood glucose(2hPG) and glycosylated hemoglobin (HbA1c) between the two groups before the intervention(p>0.05). After the intervention, the levels of these indexes in the experimental group were significantly lower than those in the control group(p=0.00) (Table-II).

**Table-II:** Comparative analysis of clinical efficacy between the two groups (*χ̅*±*S*) n=60.

Indexes		Experimental group	Control group	t	p
FBG (mmol/L)	Before intervention	11.46±3.72	11.38±3.63	0.12	0.91
	After intervention*	7.54±1.08	9.26±2.31	5.22	0.00
2hPG (mmol/L)	Before intervention	14.71±4.80	14.64±3.78	0.10	0.93
	After intervention*	8.02±1.54	10.28±2.05	6.83	0.00
HbA1c (%)	Before intervention	10.68±2.86	10.56±2.73	0.24	0.81
	After intervention*	7.52±1.87	8.90±1.34	4.65	0.00

p<0.05.

No statistically significant differences were observed in the scores of somatic function, psychological function, social function and material life status between the two groups before the intervention(p>0.05). After the intervention, the above indexes in the experimental group were significantly improved compared with those in the control group(p=0.00) (Table-III).

**Table-III:** Comparative analysis of quality of life scores of the two groups before and after the intervention (*χ̅*±*S*) n=60.

Indexes		Experimental group	Control group	t	p
Somatic function	Before intervention	46.47±6.72	46.63±6.65	0.13	0.90
	After intervention*	55.48±7.10	51.12±7.42	3.53	0.00
Psychological function	Before intervention	53.25±6.32	53.28±6.38	0.03	0.97
	After intervention*	57.60±6.25	54.81±6.62	2.87	0.00
Social function	Before intervention	58.73±8.29	58.22±8.03	0.34	0.73
	After intervention*	65.42±7.36	61.69±7.21	3.80	0.00
Material life status	Before intervention	46.25±7.67	46.32±7.39	0.05	0.96
	After intervention*	53.97±7.06	50.18±7.30	2.89	0.00

* p<0.05.

No statistically significant differences were observed in the levels of SAS and SDS between the two groups before the intervention (p>0.05). After the intervention, the levels of SAS and SDS were significantly decreased compared with those in the control group, with statistically significant differences (p=0.00) (Table-IV).

**Table-IV:** Comparative analysis of the emotional status of the two groups before and after the intervention (*χ̅*±*S*) n=55.

Indexes		Experimental group	Control group	t	p
SAS	Before intervention	63.52±7.34	63.89±7.63	0.27	0.79
	After intervention*	52.76±6.23	57.60±6.31	4.23	0.00
SDS	Before intervention	64.38±7.45	65.17±7.42	0.58	0.56
	After intervention*	50.83±6.32	55.47±6.51	3.96	0.00

* p<0.05.

The patient satisfaction in the experimental group was 92%, which was significantly higher than 78% in the control group, with a statistically significant difference (p=0.02) (Table-V).

**Table-V:** Comparative analysis of patient satisfaction between the two groups (*χ̅*±*S*) n=60.

Group	Very satisfied	Generally Satisfied	Satisfied	Uncertain	Unsatisfied	Total Satisfaction*
Experimental group	34	11	15	0	0	60 (100%)
Control group	28	12	15	2	3	55 (92%)
χ^2^						5.22
P						0.02

* p<0.05.

No statistically significant differences were observed in the levels of SOD, MDA and CAT between the two groups before the intervention (p>0.05). The levels of SOD, MDA and CAT in the experimental group were significantly better than those in the control group after the intervention, with statistically significant differences (p=0.00) (Table-VI).

**Table-VI:** Comparative analysis of oxidative stress indexes between the two groups (*χ̅*±*S*) n=60.

Indexes		Experimental group	Control group	t	p
SOD (KU/l)	Before intervention	289.57±43.72	293.56±42.06	0.52	0.61
	After intervention*	342.48±41.08	317.85±40.27	3.38	0.00
MDA (μmol/l)	Before intervention	6.73±1.30	6.68±1.25	0.21	0.83
	After intervention*	5.18±1.25	5.83±1.04	2.81	0.00
CAT (U/ml)	Before intervention	25.21±4.08	24.96±4.21	0.33	0.74
	After intervention*	34.76±7.63	28.26±6.34	5.08	0.00

* p<0.05.

## DISCUSSION

As can be seen in the results of this study, the levels of fasting blood glucose (FBG), two hours postprandial blood glucose (2hPG) and glycated hemoglobin (HbA1c) in the experimental group after the intervention were significantly lower than those in the control group(p=0.00). This can be attributed to the contribution of individualized dietary guidance to the significant improvement of patients’ health knowledge and health awareness. The quality of life of patients can be improved to some extent by providing counseling and consultation to patients, finding out the existing risk factors in time, guiding them to master personalized and rational diet methods, or maintaining patients’ emotions in a stable state. Moreover, dietary supply calculated for patients based on their economic conditions and eating habits, as well as diversified and individualized recipes more acceptable to patients, can improve their dietary compliance.^10^ Meanwhile, a rational diet intervention leads to the following effects

- Regulating lipid and blood glucose values close to or at normal values, thus correcting metabolic disorders, improving symptoms, and alleviating cardiovascular and cerebrovascular complications.^11^
- Maintaining the normal weight of patients. For those who are too thin, their energy intake should be increased to enhance their physical strength and ability to resist diseases, while for obese patients, their energy intake should be reduced and their body weight should be lowered so as to increase the sensitivity of cells to insulin and other drugs.
- Maintaining patients’ physical health to ensure normal physiological and physical activities.

As the most common type of diabetes among the elderly, Type-2 diabetes mellitus (T2DM) tends to make inroads in people aged 40-70 who are obese.^12^ In terms of its etiology, T2DM has been found to be closely related to genetic factors, lifestyle habits, and diet, among which diet is the key factor. Diet not only affects the degree of disease but also plays a crucial role in disease control.^13^ Generally, T2DM is controlled by medication, which, however, is often ineffective when used alone. There is a growing body of research revealing the vital value of diet and exercise in the treatment of T2DM.^14^ Long-term exposure to high sugar levels may affect the body’s small cells and their functions. In severe cases, abnormally high glucose levels may lead to the development of the diabetic foot, neuropathy and diabetic nephropathy. In this case, dietary intervention and exercise are recommended, which can not only reduce the intake of sugars, but also prevents abnormal elevation of glucose levels beyond the normal body load in the case of relatively low insulin secretion and insulin resistance.^15^

Comprehensive exercise is a way to promote the body’s utilization of glucose and lower the blood sugar level of patients with the help of a combination of aerobic exercise and resistance exercise.^16^ It not only prolongs the body’s uptake of glucose, but also enhances insulin sensitivity. Resistance exercise acts to increase the volume of muscle fibers and increase muscle mass for a short period of time. This induces a substantial increase in muscle uptake of glucose. Moreover, glucose uptake and utilization by muscles will continue for several hours after resistance exercise, contributing to more stable glycemic control.^17^ It was shown in this study that the levels of FPG, HbA1c, and 2hPG in the experimental group were lower than those in the control group after the intervention, suggesting the effective role of comprehensive exercise instruction combined with dietary intervention in lowering blood glucose levels and maintaining blood glucose stability in elderly patients with DM. It was revealed by Shakoor et al.^18^ that aerobic-resistance exercise, on the one hand, induces energy expenditure in the body, ameliorates the expression of oxidative stress factors and inflammation and the amount of fat accumulation, and enhances the ability of the pancreas to secrete insulin; on the other hand, it promotes the body of patients to increase the uptake of glucose by skeletal muscle.^19^ Resistance training refers to increasing insulin’s ability to transport glucose and ameliorate insulin resistance through contraction exercises of specific muscle groups.^20^ In this study, the experimental group had remarkably improved somatic function, psychological function, social function and material life status, and significantly lower levels of SAS and SDS compared to the control group after the intervention. Besides, the levels of SOD, MDA and CAT in the experimental group were significantly superior to those in the control group after the intervention. These indicate that aerobic-resistance exercise combined with dietary intervention boasts an improved level of quality of life in the treatment of elderly patients with DM by improving islet resistance and lowering blood glucose levels. During exercise, patients are able to relieve their tension and anxiety, improve their quality of life, and also significantly improve the experience of visiting the clinic, increasing their satisfaction and their awareness of the disease.^21^ Not only that, but it can also improve patients’ psychological satisfaction and security, enhance confidence in overcoming the disease, and enable them to actively cooperate with medical staff to receive treatment, which is conducive to the recovery of the disease. It was shown in our study that patient satisfaction was 92% in the experimental group, which was obviously higher than 78% in the control group (p=0.02).

### Limitations:

It includes: small sample size and short follow-up time. In response to this, more samples will be included and the follow-up time will be extended in future clinical studies, with a view to assessing the pros and cons of this intervention regimen more objectively and thus benefiting more patients.

## CONCLUSIONS

Elderly patients with Type-2 diabetes mellitus are the ones with insufficient proper knowledge of dietary control and exercise, which affects their glycemic index and the control effect of glucose-lowering drugs. In this regard, personalized dietary care intervention combined with comprehensive exercise instruction is a must for such patients, boasting benefits such as enhancing patients’ knowledge of diet and exercise and strengthening their self-management. This gives a fresh impetus to a reduction in blood glucose levels, relief of patients’ negative emotions, increased patient satisfaction and quality of life, and improved levels of oxidative stress in the body.

### Authors’ Contributions:

**XS** carried out the studies, participated in collecting data, and drafted the manuscript, and are responsible and accountable for the accuracy or integrity of the work.

**XZ** performed the statistical analysis and participated in its design.

**YC** participated in acquisition, analysis, or interpretation of data and draft the manuscript.

All authors read and approved the final manuscript.
